# Clinical pathway of COVID-19 patients in primary health care in 30 European countries: Eurodata study

**DOI:** 10.1080/13814788.2023.2182879

**Published:** 2023-03-21

**Authors:** Sara Ares-Blanco, Marina Guisado-Clavero, Lourdes Ramos Del Rio, Ileana Gefaell Larrondo, Louise Fitzgerald, Limor Adler, Radost Assenova, Maria Bakola, Sabine Bayen, Elena Brutskaya-Stempkovskaya, Iliana-Carmen Busneag, Philippe-Richard Domeyer, Dragan Gjorgjievski, Kathryn Hoffmann, Оксана Ільков, Vasilis Trifon Karathanos, Aleksandar Kirkovski, Snežana Knežević, Büsra Çimen Korkmaz, Bruno Heleno, Katarzyna Nessler, Liubovė Murauskienė, Ana Luisa Neves, Naldy Parodi López, Ábel Perjés, Davorina Petek, Ferdinando Petrazzuoli, Goranka Petricek, Bohumil Seifert, Alice Serafini, Theresa Sentker, Paula Tiili, Péter Torzsa, Bert Vaes, Gijs van Pottebergh, Shlomo Vinker, María Pilar Astier-Peña, Raquel Gómez-Bravo, Heidrun Lingner, Clinical investigators

**Affiliations:** a Federica Montseny Health Centre, Gerencia Asistencial Atención Primaria, Servicio Madrileño de Salud, Madrid, Spain; b Medical Specialties and Public Health, School of Health Sciences, University Rey Juan Carlos, Madrid, Spain; c Instituto de Investigación Sanitaria Gregorio Marañón, Madrid, Spain; d Investigation Support Multidisciplinary Unit for Primary Care and Community North Area of Madrid, Madrid, Spain; e Federica Montseny Health Centre, Gerencia Asistencial de Atención Primaria, Servicio Madrileño de Salud, Madrid, Spain; f Irish College of General Practice, MICGP, Royal College of Physician, MRCSI, Ireland; g Department of Family Medicine, Sackler Faculty of Medicine, Tel Aviv University, Tel Aviv, Israel; h Department Urology and General Practice, Faculty of Medicine, Medical University of Plovdiv, Plovdiv, Bulgaria; i Research Unit for General Medicine and Primary Health Care, Faculty of Medicine, School of Health Science, University of Ioannina, Ioannina, Greece; j Department of General Practice, University of Lille, Lille, France; k General Medicine Department, Belarusian State Medical University, Belarus; l Occupational Health Expert, Spiru Haret University, Bucharest, Romania; m School of Social Sciences, Hellenic Open University, Patra, Greece; n Medical Faculty Skopje, Center for Family Medicine, Skopje, North Macedonia; o General Practice and Primary Care, University of Vienna, Vienna, Austria; p Department of Family Medicine and Outpatient Care, Medical Faculty, Uzhhorod National University, Uzhhorod, Ukraine; q Medical Education Uni, Laboratory of Hygiene and Epidemiology, Medical Department, Faculty of Health Sciences, University of Ioannina, Ioannina, Greece; r GHS, Larnaca, Cyprus; s Faculty of Medicine, Ss. Cyril and Methodius University, Skopje, North Macedonia; t Health center Kraljevo, Kraljevo, Serbia; u Van Gürpınar District Public Hospital, Istanbul, Turkey; v Comprehensive Health Research Center, NOVA Medical School, University Nova de Lisboa, Lisboa, Portugal; w USF das Conchas, Regional Health Administration Lisbon and Tagus Valley, Lisbon, Portugal; x Department of Family Medicine UJCM, University Jagielloński, Collegium Medicum, Jagielloński, Poland; y Department of Public Health, Institute of Health Sciences, Faculty of Medicine, Vilnius University, Vilnius, Lithuania; z Imperial College London, London, UK; aa Faculty of Medicine, University of Porto, Porto, Portugal; bb Närhälsan Kungshöjd Health Centre, Gothenburg, Sweden; cc Department of Pharmacology, Sahlgrenska Academy, University of Gothenburg, Gothenburg, Sweden; dd Department of Family Medicine, University of Semmelweis, Budapest, Hungary; ee Department of Family Medicine, Faculty of Medicine, University of Ljubljana, Ljubljana, Slovenia; ff Department of Clinical Sciences in Malmö, Centre for Primary Health Care Research, Lund University, Malmö, Sweden; gg Department of Family Medicine "Andrija Stampar" School of Public Health, School of Medicine, University of Zagreb, Health Centre Zagreb West, Zagreb, Croatia; hh First Faculty of Medicine, Institute of General Practice, Charles University, Prague, Czech Republic; ii Azienda Unità Sanitaria Locale di Modena, Modena, Italy; jj Laboratorio EduCare, University of Modena and Reggio Emilia, Modena, Italy; kk Center for Public Health and Healthcare, Hannover, Germany; ll Communicable Diseases and Infection Control Unit, City of Vantaa and University of Helsinki, Helsinki, Finland; mm Department of Family Medicine, Semmelweis University, Budapest, Hungary; nn Department of Public Health and Primary Care, KU Leuven, Leuven, Belgium; pp Territorial Quality Unit, Territorial Directorate of Camp de Tarragona, Institut Català de la Salut, Health Department, Generalitat de Catalunya, GIBA-IIS-Aragón, Spain; qq Patient Safety Working Party of semFYC (Spanish Society for Family and Community Medicine) and Quality and Safety in Family Medicine of WONCA World (Global Family Doctors), Catalunya, Spain; rr CHNP, Rehaklinik, Ettelbruck. Luxembourg; ss Research Group Self-Regulation and Health. Institute for Health and Behaviour, Department of Behavioural and Cognitive Sciences. Faculty of Humanities, Education, and Social Sciences, Luxembourg University, Luxembourg, Luxembourg; tt Hannover Medical School, Center for Public Health and Healthcare, Hannover, Germany; uu Health Centre Zagreb Centar, Zagreb, Croatia; vv Department of Geriatric Medicine, Hôpitaux Robert Schuma, Luxembourg City, Luxembourg; ww Närhälsan Sannegården Health Centre, Gothenburg, Sweden; xx Department of Family Medicine "Andrija Stampar" School of Public Health, School of Medicine, University of Zagreb, Health Centre Zagreb West, Zagreb, Croatia; yy Department of Family Medicine and Outpatient Care UZHNU, Medical Faculty, Uzhgorod, Ukraine; zz Health Center Krupa na Uni, Republic of Srpska, Bosnia and Herzegovina; aaa Health Center "Dr Đorđe Kovačević", Lazarevac, Belgrade, Serbia; bbb Department of Family Medicine, Andrzej Frycz Modrzewski Krakow University, Krakow, Poland; ccc European Parliament, Luxembourg; ddd Natalija North Macedonia, Macedonia; eee Bursa Uludağ University Family Medicine Department, Turkey; fff Communicable Diseases and Infection Control Unit, City of Vantaa, Finland; ggg Department of Family Medicine and Outpatient Care, Medical Faculty, Uzhhorod National University, Uzhhorod, Ukraine

**Keywords:** COVID-19, Europe, patient care management, primary health care, standard of care, policy

## Abstract

**Background:**

Most COVID-19 patients were treated in primary health care (PHC) in Europe.

**Objectives:**

To demonstrate the scope of PHC workflow during the COVID-19 pandemic emphasising similarities and differences of patient’s clinical pathways in Europe.

**Methods:**

Descriptive, cross-sectional study with data acquired through a semi-structured questionnaire in PHC in 30 European countries, created ad hoc and agreed upon among all researchers who participated in the study. GPs from each country answered the approved questionnaire. Main variable: PHC COVID-19 acute clinical pathway. All variables were collected from each country as of September 2020.

**Results:**

COVID-19 clinics in PHC facilities were organised in 8/30. Case detection and testing were performed in PHC in 27/30 countries. RT-PCR and lateral flow tests were performed in PHC in 23/30, free of charge with a medical prescription. Contact tracing was performed mainly by public health authorities. Mandatory isolation ranged from 5 to 14 days. Sick leave certification was given exclusively by GPs in 21/30 countries. Patient hotels or other resources to isolate patients were available in 12/30. Follow-up to monitor the symptoms and/or new complementary tests was made mainly by phone call (27/30). Chest X-ray and phlebotomy were performed in PHC in 18/30 and 23/30 countries, respectively. Oxygen and low-molecular-weight heparin were available in PHC (21/30).

**Conclusion:**

In Europe PHC participated in many steps to diagnose, treat and monitor COVID-19 patients. Differences among countries might be addressed at European level for the management of future pandemics.

KEY MESSAGES

- PHC was involved in nearly all steps to detect and manage cases, initial medical care, follow-up and sick leave allocation, with differences across countries.
- Physical examination, additional complementary tests and treatments were not fully available in PHC in all countries.
- Differences among countries should be addressed at the European level to standardise the role of PHC in managing future pandemics.

## Introduction

The World Health Organisation (WHO) declared the coronavirus disease 2019 (COVID-19) a pandemic on the 11th of March 2020. Since then, there have been 267,529,236 cases in Europe, 2,143,708 deaths, by December 2022 [1]. Most COVID-19 patients were treated in primary health care (PHC) in Europe [2,3]. For instance, 85% of positive cases in Germany were treated outpatient [4], while 1565 per 100,000 patients were isolated at home in Italy in 2020 [5]. The coordinated European response has been key and epidemiological monitoring would not have been possible without case detection in primary care and secondary care. Nevertheless, it is not well-known how COVID-19 patients accessed COVID-19 medical care in Europe and which was PHC role in the pandemic disease control.

Pandemic medical care included SARS-CoV-2 detection, contact tracing, case management, treatment and monitoring in PHC. The WHO recommended home management for patients with mild or moderate symptoms if close monitoring for pneumonia could be arranged [6]. Re-organisation of PHC was necessary to attend COVID-19 patients’ consultations by suspending non-urgent visits, promoting virtual consultations, prioritising care and providing resources (personal protective equipment, hand hygiene, ventilation, technology) [7]. Moreover, special consideration was given to guaranteeing universal healthcare access and equity, particularly to vulnerable groups. This research aimed to describe PHC work scope during the COVID-19 pandemic with emphasis on similarities and differences of patient’s clinical pathways across 30 European countries.

## Methods

### Design

Cross-sectional descriptive study.

### Participants

**Figure 1.**
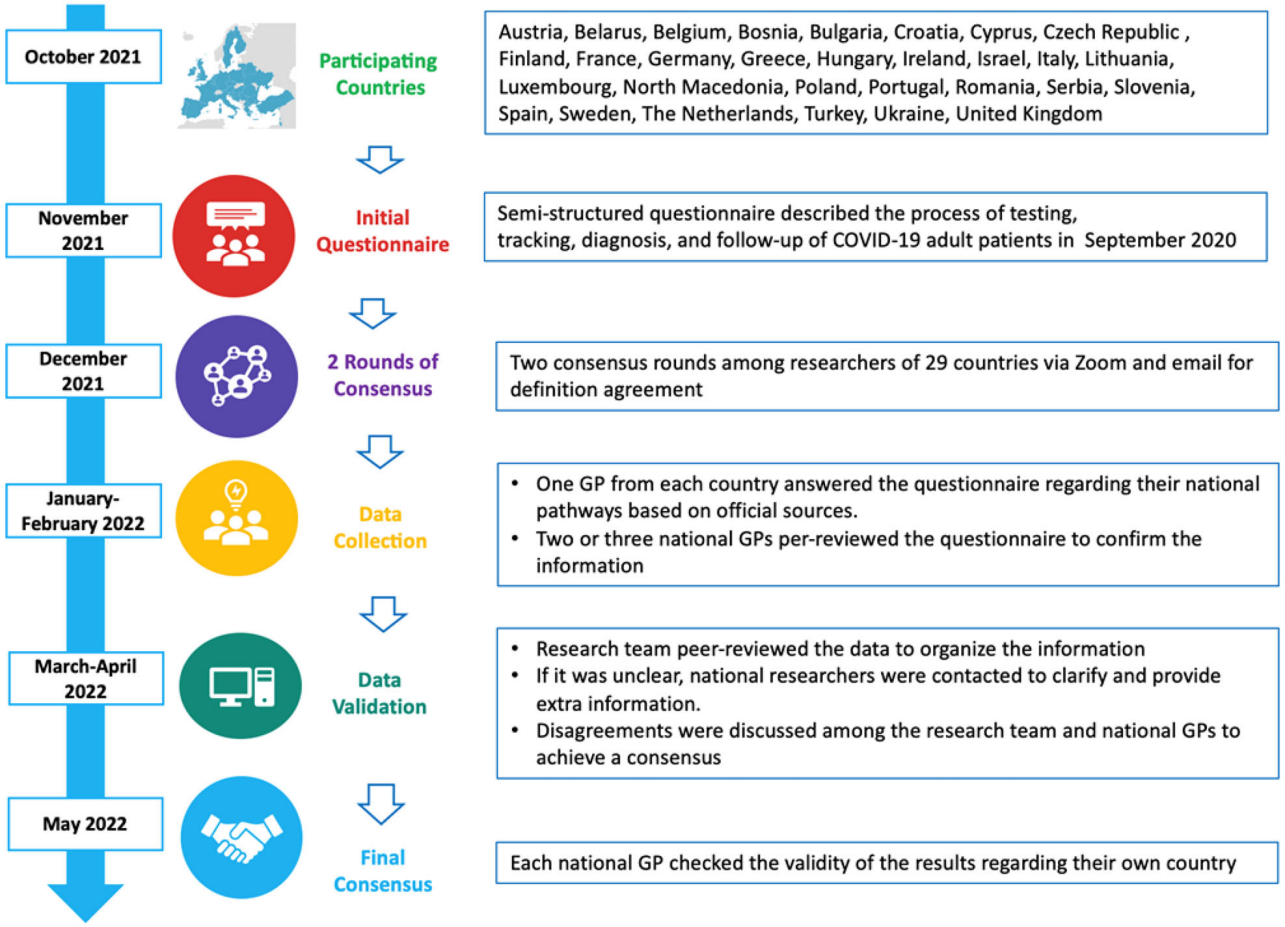
Participating countries and consensus of the questionnaire regarding the clinical pathway of COVID-19 adult patients in PHC.

In October 2021, 80 key-informants (Figure 1) were invited to participate by the World Organisation of Family Doctors (WONCA) in Europe and its networks (EGPRN and EQUIP). Information was provided by 45 GPs (42 were working clinically during the pandemic and 35 were linked to university departments), one public health expert working closely with local GPs and one medical student supervised by a participating GP. The core research team was formed by four specialists in family medicine, preventive medicine and public health.

### Questionnaire

**Figure 2.**
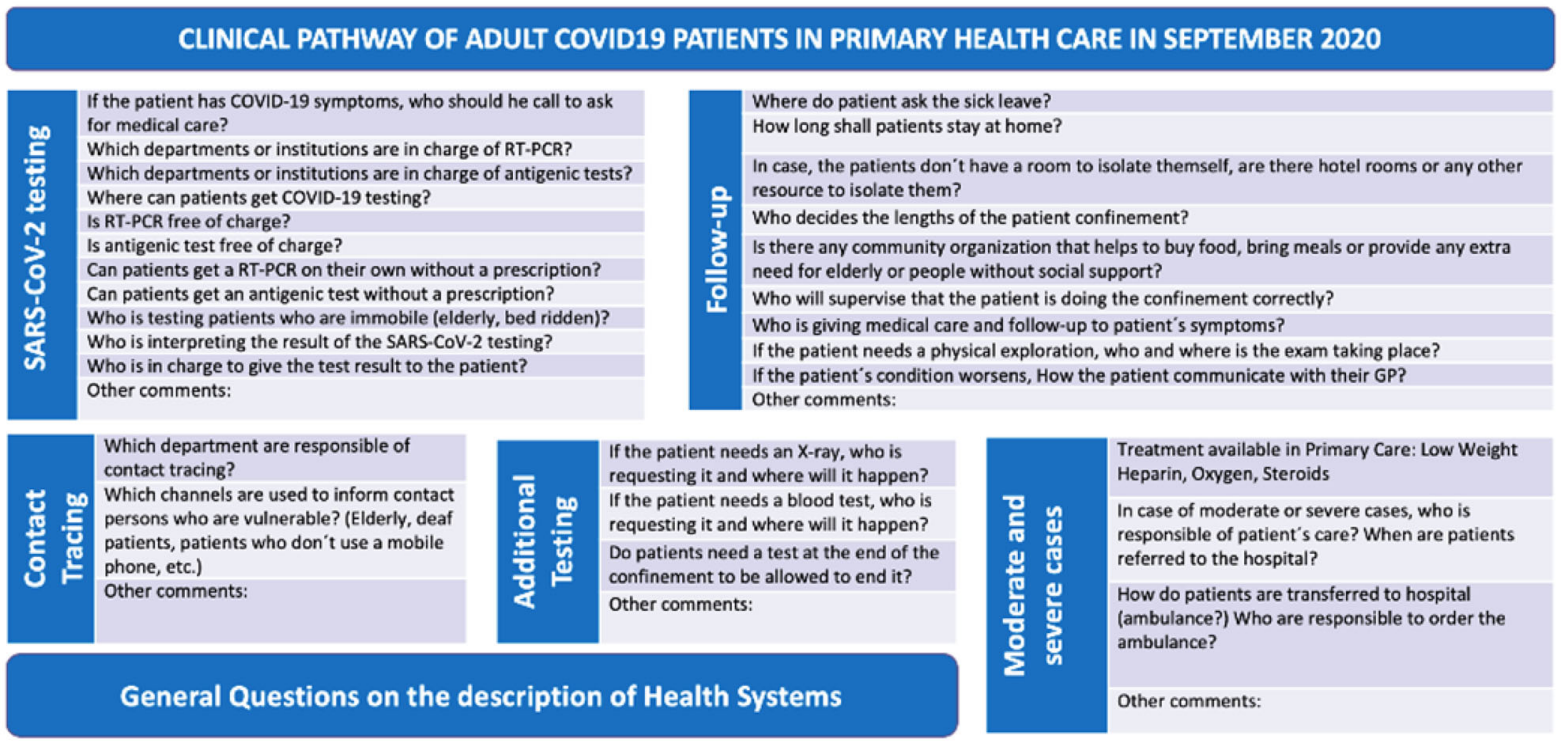
Final version of the questionnaire.

Country-specific data regarding COVID-19 outpatients’ pathways, from September 2020, was collected. The initial questionnaire was based on the WHO guidelines where PHC was involved (Figure 2 and Supplementary file 1) [6].

Three videoconferences were met to reach agreement on the final questionnaire.

Key-informants filled the semi-structured questionnaire based on official sources considered relevant and reliable (Supplementary file 2). Definitions associated with healthcare services and professionals are in Supplementary file 3.

### Data collection

At least two key-informants per country sent consensual information, after verification, regarding their national pathways implemented on September 2020.

### Data validation

The information received was checked by two core research team researchers to assure the data’s quality. If it was unclear, key-informants were contacted for clarification and to provide extra information. Disagreements were discussed among the core team and key-informants to achieve a consensus. Responses’ language was homogenised into English during data validation.

## Results

### Primary health care organisation

Different pathways to separate COVID-19 from non-COVID-19 patients in healthcare facilities were created in most countries, including special practice opening hours. Outpatient COVID-19 clinics/centres were organised in eight countries into existing PHC facilities. They provided remote assessment, testing, physical examination and some chest X-ray or phlebotomy (blood draw). In Belarus, COVID-19 centres received support from other consultants. Cyprus created a National COVID-19 department in the Ministry of Health and GPs worked 8–24 h at the Hospital COVID-19 outpatient clinic.

### Case detection and SARS-CoV-2 testing

In countries under observation, the most frequent case detection was done directly by a PHC service provider (in 27/30 countries). Additionally, in 22/30 of the countries surveyed, further services such as public health agencies, infectious diseases departments, web-based portals and/or hotlines supported suspected cases (Table 1).

**Table 1.** Initial management of COVID-19 adult patients in 30 European countries by September 2020.

Europe country	Initial medical care			SARS-CoV-2 testing		Contact tracing		Primary Health Care Information
	COVID-19 Hotline	Patient´s first contact with health system	Institution in charge of RT-PCR	Responsible for testing immobile patients	Responsible for giving test results to patients	Department responsible for contact tracing	Channels to inform vulnerable contact persons	Primary Health Care system provider
Austria	Yes	GP/Hotline	GP/Hotline	GP	Lab/GP	Local Government	Phone/Normal mailing	Mixed: Mostly Public
Belarus	Yes	GP	GP	Nurses	Nurses	State Sanitary Control Service	Nurse	Public
Belgium	Yes	GP/A&E	GP/Outpatient COVID-19 centre/ Hospital	GP	GP/Hospital/Contact tracer/Online Platform	PH/GP^$^	GP/Local health care workers	Public
Bosnia and Herzegovina	Yes	GP/Hotline	Labs	The department who takes care of the patient	SMS and in case they don’t have access: nurse phone them	PHC /PH	Phone	Public
Bulgaria	Yes	GP/A&E	PHC/PH/Hospital, Hotline	A&E/PH	The one who tested	PH	PH	Private
Croatia	Yes	GP/PH/A&E/Hotline	PH/GP	PHC/PH	Epidemiologists/ GP/ PHC nurse/ COVID-19 Hotline/ PH doctor/ PH nurse/COVID-19 clinics	PHC/PH	PH/GP	Mixed
Cyprus	No	PHC	PHC	Special units “home care” (GP and PHC nurse)	Lab/PHC/Hospital	Ministry of Health/ Department for COVID-19	Special units “home care”	Mixed
Czech Republic	No	GP	GP/Lab/Testing centre	GPs or mobile testing units*	Lab/PHC/hospital	PH	PH	Mixed
Finland	Yes	PHC/Private Sector/App	PHC/Lab	PHC/Lab	PHC	PHC	SMS/Phone and translation service	Mostly public
France	Yes	GP/Hotline	PHC/Hospital	PHC (GP/Nurse)	Lab/GP/National health insurance	National Health insurance	Direct phone calls or *via* GP	Private
Germany	Yes	GP/Hotline	PHC/PH/Mobile testing team	Mobile nursing service (PHC)/GP/ PH	RT-PHCR: GP or PH. Antigenic test: testing centre	PH	Post/ Phone/ E-mail	Private
Greece	Yes	PHC/Hotline	PHC/PH/Secondary care	PHC	GP/Internist/ PH/Lab	PH	GPs, Internists	Mixed
Hungary	No	PHC	PH	National Ambulance Services	GP	Local department of Public Health Authority	Phone/E-mail/ Family members	Public
Ireland	Yes	PHC/ Hospital	PH	Paramedic/Ambulance service	Family doctor/PH (this last one by text message)	PH	Nominated family member	Mixed
Israel	Yes	COVID-19 Telephone Hotline	PHC/A&E	COVID-19 Telephone Hotline/GP	COVID-19 Telephone Hotline, SMS	PH	Phone	Public
Italy	Yes	GP/Out of Hours	GP/Out of Hours	PHC Nurses/USCA Service	RT-PCR: SMS, EHR. Antigenic tests: Pharmacies + private laboratories + GPs	PH/App/GPs in Lombardia	GP	Public
Lithuania	Yes	PHC/Telephone Hotline/112	PHC/ Hotline	PHC	COVID-19 Telephone Hotline/GP/PHC nurse	PH	Representatives of vulnerable persons	Mixed
Luxembourg	Yes	GP/Hotline/Hospital	GP/PH	Lab came to them	Hotline/Lab/GP	PH/GP	Phone calls/Letters/ E-mails/Home visits	Mixed
Netherlands	Yes	PHC	PH	PH	PH nurses	PH	Phone/email	Public
North Macedonia	No	PHC	PH	PH	GP	PH	Phone	Public
Poland	Yes	PHC/Telephone Hotline/112	PHC/Hospital/Lab	Mobile teams (activated by PHC)	GP/Sanitary station	Sanitary stations (PH)	Sanitary stations (PH)	Mixed
Portugal	Yes	PHC/Hotline	PHC/PH	Private labs or community nurses	Lab/GP	PH	Phone calls/E-mails/family members and the outreach (social workers, PH, civil protection teams)	Mixed
Romania	Yes	GP	PH/COVID-19 ambulance/Lab	PH/COVID-19 Ambulance	Lab/PH	PH/GP	E-mail/WhatsApp/ SMS/ GP	Private
Serbia	Yes	PHC/Hotline/PH	PHC (COVID-19 Centres)	COVID-19 clinics (patients transferred by ambulance)	PHC nurse/PH nurse/ Hotline/E-health App	PH	Phone/E health	National PH Insurance Fund
Slovenia	Yes	GP/A&E	PHC	PHC	IT system (Lab or GP)	PH	PH	Mixed
Spain	Yes	PHC/Hotline	PHC/A&E	PHC (GP/Nurse)	PHC (GP/Nurse)	PHC/PH/App	PHC	Public
Sweden	No	PHC/ Hotline	PHC/A&E	PHC	Department which ordered (physicians or nurses)	Regional Infection Tracing department	PHC/ Community nurses	Mixed
Turkey	Yes	Filiation group**/ A&E	Hospital/Lab	Filiation group**	Person health account PHC	Filiation group**/GP	Filiation group**/GP	Public
Ukraine	Yes	GP	GP/Lab	Field teams	GP	PH	PH	Mixed
United Kingdom	Yes	Phone line or online platform	NHS England	Central teams	Information not available	NHS England (Test and Trace)	Information not available	Public

A&E: Accident and Emergency Department. COVID-19 centre: COVID-19 outpatient clinic where GPs are working. EHR: electronic health record. GP: General Practitioners. Lab: microbiology laboratory. PHC: Primary health care, it includes GPs, PHC nurses and other health professionals working ambulatory. PH: Public Health. RT-PCR: Reverse transcription polymerase chain reaction. SMS: short message service.

$ Double system: central system under the coordination of the three governments (Flanders, Brussels and Wallonia) combined with a local system (1:100,000 inhabitants) of contact tracing under the supervision of a local GP (medical single point of contact) *Mobile testing units were organised by different parties (municipalities, hospitals, emergency care). **doctor and nurse, driver who are assigned by Provincial Health of Infectious Diseases Department.

In all countries, RT-PCR was free in symptomatic patients and PHC was in charge, except in 8 countries. Other institutions involved were accident and emergency departments (A&E) or laboratories. In most countries, lateral flow test was also free but not available in seven countries by September 2020. It was mainly used in PHC and other services such as pharmacies or ambulances.

Testing was performed simultaneously in several places in most countries (PHC facilities, certified microbiology laboratories, public health institutions, hospitals or pharmacists for lateral flow tests). However, for immobile patients, community nurses or primary care home units were primarily the services acquiring SARS-CoV-2 samples. Sometimes, microbiology laboratories and ambulance services were involved (Table 1).

### Administrative case management

Information regarding health systems and PHC organisation is described in Supplementary file 1.

*Case investigation and contact tracing* was part of public health services in all countries, delivered partly or entirely by PHC in Bosnia and Herzegovina, Croatia, Finland, Spain and Turkey (Table 1). Isolation of COVID-19 patients was mandatory in all countries. The duration was generally 14 days (18 countries), followed by 10 days (9 countries). COVID-19 patients had to be isolated two or three days without symptoms and in Belarus, Czech Republic and Ukraine until having a negative test (Table 2).

**Table 2.** Description of isolation and follow-up in 30 European countries by September 2020.

Europe country	Isolation		Patient’s Follow-up			Additional testing in Primary Health Care		Primary Health Care Information
	Length of isolation	Supervision of the isolation	Responsible for the sick leave	Responsible for the patient´s follow-up	Responsible for physical examination and place	Chest X-ray performance	Phlebotomy performance	Restrictions to treatment prescription
Austria	10 days if asymptomatic If symptomatic, till improvement	PH	Day 1–10: PH Day ≥ 11: GP	GP	GP at home visit	Hospital	GP: Home visit	LMWH^#^, Oxygen
Belarus	14 days + IgM/IgG testing	Police	GP /Infectious Disease specialist	GP	GP at COVID-19 Centre	COVID-19 centre	COVID-19 centre	No
Belgium	10 days if asymptomatic If symptomatic, till improvement	Police	GP	GP	GP (include home visits)/Hospital if severe cases	GP	GP	No
Bosnia and Herzegovina	14 days if 3 days asymptomatic	Police/Sanitary inspection	GP	GP	GP at COVID-19 Centre	PHC/Hospital	GP/Secondary Care	No
Bulgaria	14 days	PH/Police	GP	GP/A&E	A&E at home/ Hospital	Hospital	Hospital	No
Croatia	14 days if 3 days asymptomatic	Civil Defence/PHC	GP	PHC	PHC /COVID-19 centre/COVID-19 Hospital	PHC/COVID-19 Hospital/A&E	PHC/COVID-19 centre COVID-19 Hospital	LMWH^#^, antiviral, oxygen
Cyprus	14 days	GP	GP	GP	GP at COVID-19 centre/ COVID-19 Hospital	COVID-19 centre/ COVID-19 Hospital	COVID-19 centre/ COVID-19 Hospital	No
Czech Republic	7 days + Negative RT-PHCR	Nobody	GP	GP	GP at PHC/Home visit	PHC	GP: Home visit	No
Finland	14 days	PHC	PHC	PHC	PHC/PH/A&E	PHC/PH/A&E	PHC: Health centre	No
France	14 days	Nobody	GP/ Online self-certified	PHC	GP at Home visit	Hospital	PHC: Home visit	Oxygen
Germany	10 days	PH/Police	GP	GP	GP at Home visit	PHC/Hospital	PHC: Home visit	No
Greece	14 days	PH/Police	PHC/Secondary care	GP/Internist	GPs/Internists at home visit/PHC	PHC/Hospital	PHC/Hospital	No
Hungary	10 days if 3 days asymptomatic	Police	GP	GP	Hospital	Hospital	Hospital	LMWH^ and antibiotics^^
Ireland	14 days if 5 days without fever	Nobody	GP	PHC	PHC/ A&E	A&E	A&E	No
Israel	14 days	Police	GP	COVID-19 Hotline	Special PHC unit at home visits/ Hospital	A&E	A&E	No
Italy	14 days + Negative RT- PCR 21 days without RT-PCR testing	PH	GP	PHC	GP at PHC/home visit	Hospital	COVID-19 centre/ Hospital	No
Lithuania	14 days	PH/Police	PHC	GP	PHC at home visit	PHC/ COVID-19 centre /Hospital	PHC/ COVID-19 centre	No
Luxembourg	14 days	PH	GP	GP/PH (follow up platform)	COVID-19 centre/ A&E	Hospital	Hospital	No
Netherlands	7–14 days with 24h asymptomatic	No supervision	No sick leave needed^##^	PHC	GP	GP	GP	No
North Macedonia	10 days if asymptomatic 20 days if symptomatic	Police	GP	GP/Infectious disease specialist	GP/Hospital	PHC/Hospital	PHC/Hospital	Oxygen
Poland	14 days	Police/Army	Automatically with a positive test/PHC	PHC/Hospital (hospitalised patients)	PHC/Home visit/Hospital	GP/PHC/Hospital	GP/PHC/Hospital	No
Portugal	14 days	PHC/PH/Hotline	GP: outpatients Hospital: inpatients	GP/Hospital	PHC, A&E (depending on severity)	Hospital	Hospital	No
Romania	14 days	PH/Ambulance**	GP	GP/Rescue Services	Ambulance** at home	COVID-19 centre	COVID-19 hospital	Heparin
Serbia	14 days if 3 days asymptomatic	Police/Sanitary inspectors/Internal Affairs Ministry	GP	GP/Hotline PHC: COVID-19 centre	GP at COVID-19 centre	PHC	COVID-19 centre	LMWH^ and antibiotics^^
Slovenia	10 days if 2 days asymptomatic	PHC	GP	PHC	COVID-19 centre	A&E	COVID-19 centre	Oxygen
Spain	10 days if 3 days asymptomatic	PH/PHC	GP	PHC	GP at PHC/Home visit	PHC	PHC (Health centre or home visit)	No
Sweden	7 days	Nobody	Day 1–21: no needed Day ≥ 22: GP	PHC	GP at PHC	A&E	PHC	No
Turkey	10 days if asymptomatic 14 days if hospitalised 20d if ICU admission	Filiation group***/GP	GP	GP: Phone calls Infectious Disease doctor: Home visit	COVID-19 centre/ A&E/Hospital/ COVID-19 hospital	COVID-19 centre/Hospital	COVID-19 centre/ COVID-19 Hospital	No
Ukraine	Until negative RT-PCR	PH	GP	GP	GP at PHC	PHC	PHC/Private Labs: Home visit	Oxygen
United Kingdom	10 days	NHS Test and Trace	Day 1–7: self-certified GP subsequently	NHS England	PHC, A&E (Depending on severity)	Hospital	PHC/Hospital	No

A&E: Accident and Emergency Department. COVID-19 centre: COVID-19 outpatient clinic where GP are working. COVID-19 Hospital: Hospital dedicated exclusively or mainly to COVID-19 patients. GP: General Physician. Hotline: COVID-19 hotline telephone. ICU: Intensive Care Unit. LMWH: Low-molecular-weight heparin. PHC: Primary Health Care, it includes GP, PHC nurses and other health professionals working ambulatory. PH: Public Health. RT-PCR: Reverse transcription polymerase chain reaction.

*Patients who have not registered with a GP are attended at USCA. USCA is a Special Unit of Out of Hour Service.

**Ambulance: Ambulances depends on Rescue Services in Rumania.

***Infectious disease doctor works along a nurse and a driver to do home visits. They depend on the Infectious Disease Department.

^#^GPs could prescribe these treatments under the supervision of a hospital consultant and if complies with professional guidelines.

^##^People do not have to ask for sick leave. When people are sick, they mention it to their employer and there is no statement of a doctor required.

^LMWH was not subsidised under GPs prescription.

^^Antibiotics in case of coexisting bacterial infection were not subsidised under GPs prescription.

*Paid sick leave* was exclusively managed by GPs in 21 countries. Other healthcare professionals, such as members of infectious disease departments, doctors in secondary care or public health departments helped to process them too. It was automatically set after a positive test in Poland. Only France allowed self-declaration for work absenteeism or GPs’ sick note, and the United Kingdom permitted self-certified leave declarations for the first seven days of diseases. Sweden did not demand any sick leave until day 22 of the disease. In the Netherlands, sick leave was not required either; patients mentioned it to their employer without doctor’s statements.

*Social support* became vital during isolation to guarantee basic needs. Social services provided care in 25 countries and charities gave support in most of them, in collaboration with social services. The Ministry of Health of Serbia created a website with volunteers available to facilitate the contact for those in need. In Croatia, public institutions (Ministry of Labour and Welfare, Red Cross) published a list of different volunteers/NGOs. The possibility of offering a hotel room or other resources for those who could not isolate at home was described in 11 countries. Lithuania offered beds at the municipalities.

### Clinical case management

In all countries, patients’ follow-up was made by PHC through phone calls. E-mail or video consultations were available in some places (Supplementary file 3). Outpatients were followed in PHC to check the symptoms’ evolution, social support requirement and need for additional testing. This process was carried out exclusively in PHC in 19/30 countries. Follow-up was also shared with other specialists, including A&E doctors, infectious disease doctors and internists. If patients needed physical examination, it was performed at PHC in 27 countries, including home visits. Chest X-ray (18/30 countries) and phlebotomy (23/30 countries) were available in PHC. Patients were referred to hospitals if symptoms were worsening (Table 2).

Ambulatory treatments, including low-molecular-weight heparin and oxygen could be prescribed by PHC in 21/30 countries. In Croatia and Serbia, GPs could only prescribe low-molecular-weight heparin after hospital specialists’ recommendation and/if it complied with professional guidelines. In Hungary, low-molecular-weight heparin was not reimbursed if the prescription was from PHC.

## Discussion

### Main findings

This study describes PHC role in managing COVID-19 patients in 30 European countries. PHC was involved in nearly all steps of detection and case management, from initial medical care to diagnose, follow-up and sick leaves with varying practices across countries. Public health authorities were involved in contact tracing and, in some countries, also in testing organisation and result reporting. The length of isolation ranged from 5 to 14 days. Physical examination, additional examinations and treatment were available in most countries; however, a few countries lacked some specific interventions.

### Strengths and limitations

A description of disease control pathways in the COVID-19 pandemic in different European countries has not been written before. The information was collected from publicly available reliable online resources by local researchers. They were working in PHC or in close touch with GPs describing how pathways were adapted in real practice. Changes of the pathways could have happened in some regions because of the workload of cases. Although key-informants answered the questionnaires from publicly available trusted network resources, not all relevant information may have been found. In Sweden, the information is from Västra Götaland region, and in United Kingdom, the information is from England. There were not key-informants in other regions. As the health care systems in Europe vary, the direct comparison of practices was not possible; however, we describe similarities. The different solutions described in this study may inspire other countries to adapt them to their needs.

### Comparison with existing literature

A study from the United States reported that COVID-19 hotlines referred 42% of calls to a physician and of those assessed, self-isolation was recommended to 79% of the cases [8]. In this study, 12 countries launched a hotline for access to medical assessment of suspected cases. Although, telemedicine was prioritised during the pandemic, only Finland developed a web-based portal to facilitate access to medical assessment. Most mobile applications were not connected with PHC [9]. In our study, few countries developed online tools to improve the care of patients in PHC, although most patients were attended there. COVID-19 testing was mainly carried out in PHC while public health agencies were in charge of tracking. However, COVID-19 data gathered by administrations, nationally and internationally, overlooked that PHC has been the first line of medical care [10,11].

The Ministry of Health of all participating countries facilitated the accessibility of COVID-19 testing by funding the fees when it was prescribed, which was in line with the principle of universal healthcare access and the coordinated WHO pandemic response. Testing was based on RT-PCR tests in all the countries, but lateral flow testing was not available in any by September 2020. Advantages of testing was based on its price, transportability, possibility of self-managing and quick results [12]. COVID-19 testing varied through countries depending on the institution in charge of the test (PHC or public health), accessibility and affordability of tests, sensibility and specificity of tests [13].

The transmission of the SARS-CoV-2 was more frequent in the first 5 days; however, the incubation could extend until day 15 [14]. The criteria for discharging patients from isolation required three days without symptoms but the length differed from 8 days (European Control of Disease Centre) to 10 days (WHO) [14,15]. There was a remarkable lack of homogeneity in the length of isolation and protocols for ending it in Europe. Isolation is an element of pandemic control; 18 countries decided longer isolation (14 days or more) against the health institution’s recommendation. More resilient health systems responded comprehensively with multi-ministry task forces [16]. The lack of a common message among European countries could hinder compliance with isolation rules [17].

In the first wave of the pandemic, sick leave for respiratory diseases nearly doubled the number of cases in the same period during 2017–2019 (4.9 cases/1000 workers vs 2.5 cases/1000 workers) [18]. Other reported data showed that 62.2% of COVID-19 patients needed sick leave in Germany and in Sweden, the median duration was 35 days [19,20]. Well-designed paid sick leave is critical to ensure workers stay home to prevent the spread of SARS-CoV-2 and other infectious pathogens, both when the economy is open and during shutdowns. A GP sick leave certificate was needed in most countries, mainly managed by GPs in very crowded practices [21]. France, Sweden and United Kingdom allowed self-reported paid sick leave while the Netherlands did not require sick leave certificate when getting sick, which might reduce the work overload for GPs. It is crucial to prioritise GPs’ time in activities that add value to patient´s care as well as reduce the inverse care law [22].

We highlight the role of GPs in the management of COVID-19 patients. PHC had a significant role in clinical case management in all countries and some countries had restrictions on medical assessment and treatments. First, it will be relevant for European countries to invest in practices to guarantee safe settings to care for airborne infectious diseases, perhaps through the accreditation of PHC practices as in Denmark [23]. Second, as symptoms are not enough to diagnose COVID-19 or identify severe cases, there is a need to examine and perform chest X-ray to rule out pneumonia in PHC. Studies that analysed pathways in other countries did not describe the use of additional testing [24]. Moderate pneumonia could be managed in PHC if phlebotomy was accessible and treatment possible [25,26]. Restrictions in COVID-19 treatment in PHC or induced prescription by other specialists is inconsistent with evidence-based medicine [6]. In September 2020, there was evidence of the benefit of heparin [27], thus not allowing PHC practitioners to prescribe this or oxygen, reduced the management capacity of PHC [28], as well as, not respecting some patients’ wish to be treated at home [29,30]. These restrictions may have unnecessarily hindered the effective outpatient care and pushed patients to hospitals. Therefore, it could be beneficial to study opportunities to increase diagnosing and treatment capacity of PHC during pandemics.

### Implications for research and/or practice

This study showed that PHC has a significant role in COVID-19 disease control and management in most European countries, as it takes up PHC resources and may affect the ability to deliver other services. It also requires specific skills, equipment and flexibility to reorganise services. Therefore, the burden of communicable disease outbreaks for PHC should be recognised, monitored and supported by additional resources. Self-reported paid leave should be simplified during pandemics to reduce bureaucracy and GPs workload. At European level, there are three crucial needs for future pandemics: (1) a common guidance and implementation of the isolation period within Europe; (2) a legislation to reduce the bureaucracy of sick leave certification in PHC and, (3) the implementation of a European Primary Care Information System linked to the European Centre for Disease Prevention and Control (ECDC).

## Conclusion

In Europe, PHC was involved in most steps of COVID-19 medical care in the community, from the suspected cases to diagnosis and follow-up. Inequalities in the access to physical examination, complementary tests and treatments were found. These differences might be addressed through the implementation of European PHC recommendations. Future pandemics must have a Europe common agreement.

## Supplementary Material

Supplemental Material

Supplement 3

Supplement 2

Supplement 1

## Clinical investigators

Asja Ćosić Divjak^uu^, Maryher Delphin Peña^vv^, Mila Gómez-Johansson^ww^, Miroslav Hanževački^xx^, Shushman Ivanna^yy^, Marijana Jandrić-Kočić^zz^, Milena Kostić^aaa^, Anna Krztoń-Królewiecka^bbb^, Martin Sattler^ccc^, Natalija Saurek-Aleksandrovska^ddd^, Canan Tuz Yilmaz^eee^, Kirsi Valtonen^fff^ and Kaliy Vasyl^ggg^

^uu^Health Centre Zagreb Centar, Zagreb, Croatia

^vv^Department of Geriatric Medicine, Hôpitaux Robert Schuma, Luxembourg

^ww^Närhälsan Sannegården Health Centre, Gothenburg, Sweden

^xx^Department of Family Medicine "Andrija Stampar" School of Public Health, School of Medicine, University of Zagreb, Croatia. Health Centre Zagreb West, Croatia.

^yy^Department of Family Medicine and Outpatient Care UZHNU, Medical Faculty, Ukraine.

^zz^Health Centre, Krupa na Uni, Republic of Srpska, Bosnia and Herzegovina.

^aaa^Health Centre "Dr Đorđe Kovačević", Lazarevac, Belgrade, Serbia.

^bbb^Department of Family Medicine, Andrzej Frycz Modrzewski Krakow University, Krakow, Poland

^ccc^European Parliament, Luxembourg, Luxembourg.

^ddd^Faculty of Medicine, University of Sv. Kiril I Metodij, Skopje, Republic of North Macedonia

^eee^Bursa Uludağ University Family Medicine Department, Bursa, Turkey.

^fff^Communicable Diseases and Infection Control Unit, City of Vantaa, Vantaa, Finland.

^ggg^Department of Family Medicine and Outpatient Care, Medical Faculty, Uzhhorod National University, Ukraine
