# Supplementary material for: Clinical pathway of COVID-19 patients in primary health care in 30 European countries: Eurodata study
Source: Eur J Gen Pract. 2023 Mar 21;29(2):2182879. doi: 10.1080/13814788.2023.2182879 (PMC10324993; doi:10.1080/13814788.2023.2182879)
Supplement: Supplemental Material [file IGEN_A_2182879_SM7888.pptx]

## Slide 1
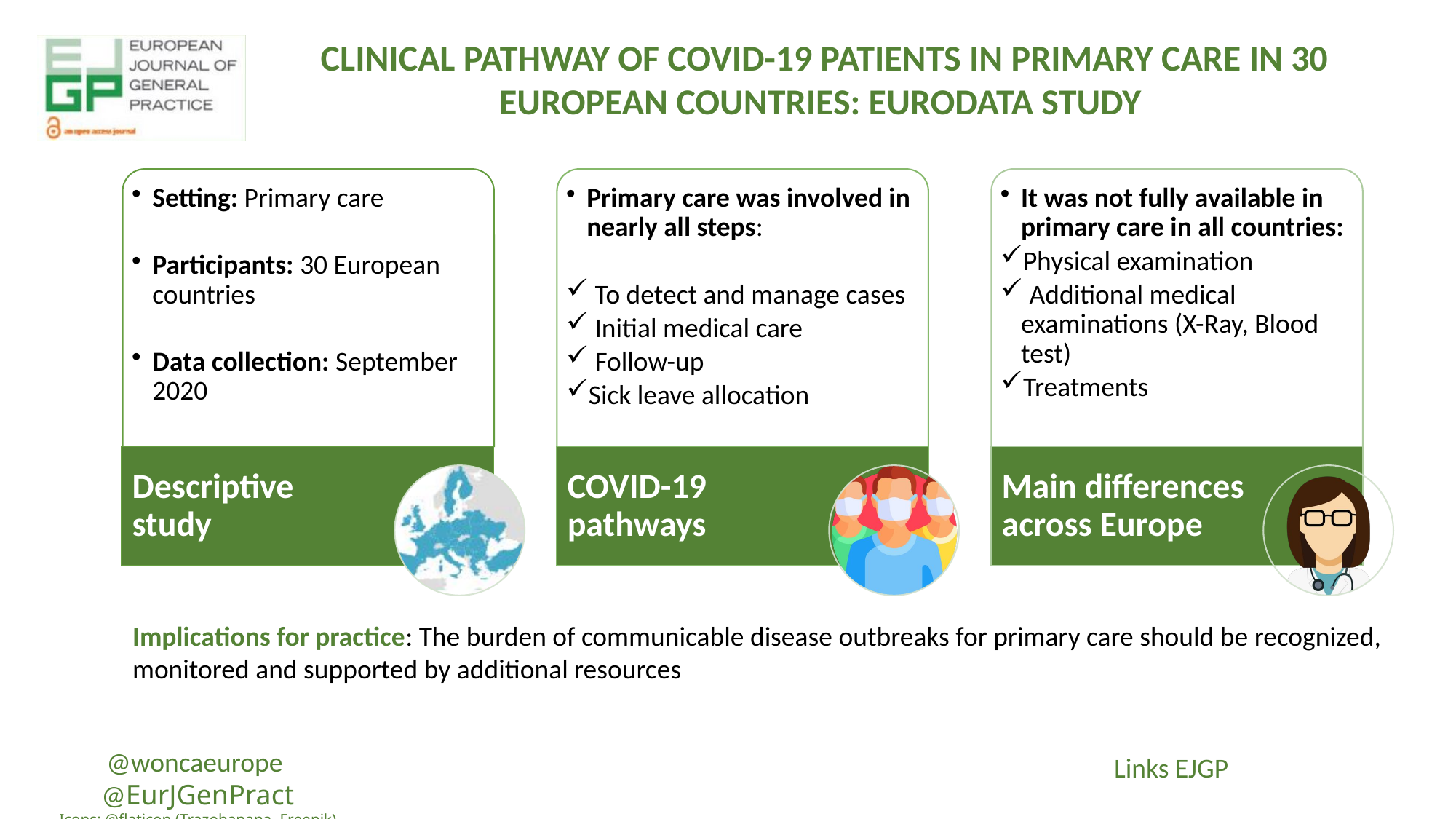

CLINICAL PATHWAY OF COVID-19 PATIENTS IN PRIMARY CARE IN 30 EUROPEAN COUNTRIES: EURODATA STUDY
Implications for practice: The burden of communicable disease outbreaks for primary care should be recognized, monitored and supported by additional resources
@woncaeurope @EurJGenPract
Icons: @flaticon (Trazobanana, Freepik)
Links EJGP
