# Supplementary material for: Clinical pathway of COVID-19 patients in primary health care in 30 European countries: Eurodata study
Source: Eur J Gen Pract. 2023 Mar 21;29(2):2182879. doi: 10.1080/13814788.2023.2182879 (PMC10324993; doi:10.1080/13814788.2023.2182879)
Supplement: Supplement 3 [file IGEN_A_2182879_SM3845.docx]

**Supplement 3.** Definitions of each service and healthcare professional in this study. The definitions have been adapted to the COVID-19 pandemic from the MeSH terms in PubMed:

- **Primary care:** Integrated and accessible health care services provided by a general practitioner (GP) or a primary care nurse in the context of family and community.
- **A&E:** Accident & Emergency department or Emergency Department. Hospital department responsible for the administration and provision of immediate medical to the COVID-19 patient.
- **COVID-19 Telephone Hotline:** A direct communication system, usually telephone, established for instant contact. It is designed to provide only information about COVID-19 and assistance through trained personnel and is used for counselling and referrals.
- **Clinical Pathways**: Schedules of medical procedures, including diagnostic tests, medications, and consultations designed to affect an efficient, coordinated treatment programme for COVID-19 patients.
- **EGPRN:** European General Practice Research Network working group from WONCA Europe.
- **EQUIP**: European Society for Quality and Safety in Family Medicine of WONCA Europe.
- **Hotline:** A direct communication system, usually telephone, established for instant contact. It is designed to provide special information and assistance through trained personnel and is used for counselling, referrals, and emergencies such as poisonings and threatened suicides.
- **Public Health:** Public health refers to all organised measures (whether public or private) to prevent disease, promote health, and prolong life among the population. Its activities aim to provide conditions where people can be healthy and focus on entire populations, not individual patients or diseases. Thus, public health is concerned with the total system and not only the eradication of a particular disease (WHO definition: <https://www.euro.who.int/__data/assets/pdf_file/0007/152683/e95877.pdf>).
- **GP:** General practitioner or family doctor. Physicians who are responsible for the provision of comprehensive and continuing care to every individual seeking medical care irrespective of age, sex and illness and they care for individuals in the context of their community (WONCA Europe definition: https://www.woncaeurope.org/page/definition-of-general-practice-family-medicine).
- **LFTI (Lateral Flow test immunoassay):** It is a technique that uses antibodies for identifying or quantifying a substance. Usually, the substance being studied serves as antigen both in antibody production and in measurement of antibody by the test substance.
- **Phlebotomy:** It is the technique used to draw blood from a vein for diagnostic purposes or for treatment of certain blood disorders such as erythrocytosis, hemochromatosis, polycythemia vera, and porphyria cutanea tarda.
- **Primary care nurse:** Nurses who provide care to patients of all age levels, and who focus their efforts on the health care needs of the entire family in the community context.
- **Primary Health Care**: Integrated, accessible health care services provided by a general practitioner (GP) or primary health care nurse who are accountable for addressing a large majority of personal health care needs, developing a sustained partnership with patients, and practising in the context of family and community.
- **Public Health doctor:** Doctor whose goal is to improve health and quality of life in a population or community through preventing and treating diseases, the surveillance of cases and health indicators, and promoting healthy behaviours through public education and awareness.
- **Public Health nurses**: Nurses whose goal is to improve health and quality of life in a population or community through the prevention and treatment of diseases, the surveillance of cases and health indicators, and the promotion of healthy behaviours through public education and awareness.
- **RT**-**PCR:** Reverse Transcriptase Polymerase Chain Reaction, A variation of the PCR technique in which cDNA is made from RNA via reverse transcription. The resultant cDNA is then amplified using standard PCR protocols.
- **Scope**/**the scope of practice**: The services licensed professionals are deemed competent to perform and permitted to undertake in keeping with the terms of their professional license.
- **Social Services:** The use of community resources, individual casework, or group work to promote the adaptive capacities of individuals in relation to their social and economic environments.
- **WONCA:** World Organization of Family Doctors.
