# Supplementary material for: Clinical pathway of COVID-19 patients in primary health care in 30 European countries: Eurodata study
Source: Eur J Gen Pract. 2023 Mar 21;29(2):2182879. doi: 10.1080/13814788.2023.2182879 (PMC10324993; doi:10.1080/13814788.2023.2182879)
Supplement: Supplement 1 [file IGEN_A_2182879_SM3843.docx]

**Supplement 1.** General information regarding the health system and how Primary Health Care was organised in 30 Europe countries during COVID-19 pandemic

| COVID-19 telephone hotline |  |  |
| --- | --- | --- |
|  | Yes | Austria, Belarus, Belgium, Bosnia and Herzegovina, Bulgaria, Croatia, Finland, France, Germany, Greece, Ireland, Israel, Italy, Lithuania, Luxembourg, Netherlands, Portugal, Romania, Serbia, Slovenia, Spain, Turkey, Ukraine |
|  | No | Cyprus, Czech Republic, Hungary, North Macedonia, Sweden |
| Healthcare professionals in PHC (most frequent professionals) |  |  |
|  | GP | Austria, Belarus, Belgium (including a local GP who coordinates the contact tracing), Bosnia and Herzegovina, Bulgaria, Croatia, Cyprus, Czech Republic, Finland, France, Germany, Greece, Hungary, Ireland Israel, Italy, Netherlands, North Macedonia, Lithuania, Luxembourg, Portugal, Romania, Serbia, Slovenia, Spain, Sweden, Turkey, Ukraine |
|  | Nurse | Austria, Belarus, Belgium, Bosnia and Herzegovina, Bulgaria, Croatia, Cyprus, Czech Republic, Finland, France, Greece, Hungary, Ireland, Israel, Italy, Netherlands, North Macedonia, Lithuania, Luxembourg, Portugal, Romania, Serbia, Slovenia, Spain, Sweden, Turkey, Ukraine |
|  | Home care nurses | Belgium, Cyprus, Czech Republic, Finland, Luxembourg, Netherlands, Spain |
|  | Midwife | Belgium, Finland, France, Greece, Hungary, Italy, Netherlands, Spain, Sweden |
|  | Physiotherapist | Austria, Belgium, Cyprus, France, Israel, Italy, Luxembourg, Portugal, Slovenia, Spain, Sweden. |
|  | Social worker | Austria, Belgium, Israel, Cyprus, France, Greece, Luxembourg, Netherlands, Portugal, Slovenia, Spain, Sweden |
|  | Psychotherapist | Austria, Belgium, France, Luxembourg, Netherlands, Portugal, Sweden |
|  | Paediatrician | Belarus, Belgium, Croatia, Cyprus, Czech Republic, France, Germany, Greece, Hungary, Israel, Italy, Luxembourg,  Romania, Serbia, Slovenia, Spain |
|  | Dentist | Belgium, Croatia, Finland, Greece, Hungary, Lithuania, Luxembourg, Slovenia, Spain, Portugal |
|  | Secretary | Austria, Belgium, Greece, Israel, Luxembourg, Sweden, Portugal, Spain |
|  | Other specialties* | Austria, Belgium, Croatia, Czech Republic, Finland, France, Greece, Italy, Lithuania, Luxembourg, Portugal, Romania, Serbia, Spain, Sweden, Turkey |
| *Other specialities include dieticians, epidemiologist, cardiologist, endocrinologists, gynaecologists, internists, pulmonologists, mental health professionals, work doctor. | | |
| Remote assessment in primary care (phone, video consultation, email) |  |  |
|  | Phone | Austria, Belarus, Belgium, Bosnia and Herzegovina, Bulgaria, Croatia, Cyprus, Greece, Luxembourg, Netherlands, Portugal, Romania, Serbia, Slovenia, Spain, Sweden, Turkey, Ukraine |
|  | Video consultation | Austria, Belgium, Greece, Luxembourg, Netherlands, Romania, Slovenia, Sweden |
|  | Email | Austria, Croatia, Luxembourg, Portugal, Slovenia |
|  | Not specified | Czech Republic, Hungary, Finland, France, Germany, Ireland, Israel, Italy, Lithuania, North Macedonia |
| Face to face appointments to explore COVID-19 patients in primary care |  |  |
|  | Yes | Austria, Belarus, Belgium, Bosnia and Herzegovina, Bulgaria, Croatia, Czech Republic, Finland, France, Germany, Greece, Ireland, Lithuania, Luxembourg, Netherlands, North Macedonia, Portugal, Romania, Serbia, Slovenia, Spain, Sweden, Ukraine (not frequent). |
|  | No | Cyprus, Hungary, Israel, Italy, Turkey |

| Range of age |  |  |
| --- | --- | --- |
|  | All ages | Austria, Belgium, Bulgaria, Croatia, Finland, France, Greece, Hungary, Ireland, Israel, Lithuania, Luxembourg,  Netherlands, North Macedonia, Portugal, Romania, Sweden, Serbia, Turkey, Ukraine |
|  | > 5 years old | Bosnia and Herzegovina^ Germany, |
|  | > 14 years old | Cyprus^, Czech Republic, Italy^, Spain |
|  | > 18 years old | Belarus, Slovenia |
| ^Bosnia and Herzegovina over 6, Cyprus over 15, Italy over 6 optional |  |  |
| Primary healthcare system provider |  |  |
|  | Public | Belarus*,* Belgium, Bosnia and Herzegovina, Finland, Hungary, Israel, Italy, Netherlands, North Macedonia, Serbia, Turkey |
|  | Private | Bulgaria, France, Germany, Romania |
|  | Mixed | Austria#, Cyprus, Croatia, Czech Republic, Greece #, Finland #, Ireland, Lithuania, Luxembourg, Portugal #, Slovenia #, Spain #, Sweden, Ukraine |
| #These countries specify that there is a public system for all citizens but you can as well go to private clinics. In Portugal 30% population has access to private insurance, in Slovenia 20% are private contractors | | |
| Employment status in your country |  |  |
|  | Salaried with the government | Belarus, Bosnia and Herzegovina, Bulgaria, Croatia, Cyprus, Finland, Germany, Greece, Lithuania, Portugal,  Serbia, Slovenia, Spain, Sweden, Turkey, Ukraine |
|  | Self-employed with contract to health insurance fund(s) or health authority | Austria, Bulgaria, Croatia, Cyprus, Czech Republic, Finland, France, Germany, Greece, Hungary, Ireland, Israel, Italy,  Lithuania, Luxembourg, Netherlands, North Macedonia, Portugal, Romania, Slovenia, Spain, Sweden, Ukraine |
|  | Self-employed without contract (paid by patients out-of-pocket) | Austria, Belgium, Croatia, Finland, Germany, Greece, Ireland, Spain |
| Description of how primary healthcare professionals’ salary is calculated |  |  |
|  | Payment by medical act (consultation, home visit, etc.) | Austria, Belgium, Bulgaria, Croatia, Czech Republic, France, Germany, Greece, Ireland, Israel, Italy,  Lithuania, Luxembourg, Netherlands, Portugal, Romania |
|  | Payment by the number of patients attended | Austria, Croatia, Cyprus, Germany, North Macedonia, Ireland, Italy, Israel, Turkey, Ukraine |
|  | Payment by capitation | Belgium, Bulgaria, Czech Republic, Hungary, Lithuania, Netherlands, Poland, Portugal, Romania, Serbia, Ukraine |
|  | Fixed salary | Belarus, Belgium, Bosnia and Herzegovina, Croatia, Czech Republic, Finland, Greece, Hungary, Israel,  Lithuania, Portugal, Serbia, Slovenia, Spain, Sweden |
|  | Bonus/supplements | Belarus, Bulgaria, Czech Republic, Finland, France, Hungary, Israel, Lithuania, Spain |
|  | | |
